# Coupled Biomechanical Response of the Cornea Assessed by Non-Contact Tonometry. A Simulation Study

**DOI:** 10.1371/journal.pone.0121486

**Published:** 2015-03-17

**Authors:** Miguel Á. Ariza-Gracia, Jesús F. Zurita, David P. Piñero, José F. Rodriguez-Matas, Begoña Calvo

**Affiliations:** 1 Aragón Institute of Engineering Research (I3A), University of Zaragoza, Zaragoza, Spain; 2 Department of Mechanical Engineering, Energetics and Materials, Public University of Navarra, Pamplona, Spain; 3 Ophthalmology Department (OFTALMAR), Medimar International Hospital, Alicante, Spain; 4 Optics, Pharmacologist and Anatomy Department, University of Alicante, Alicante, Spain; 5 Bioengineering, Biomaterials and Nanomedicine Online Biomedical Research Center (CIBER-BBN), Zaragoza, Spain; Tel Aviv University, ISRAEL

## Abstract

The mechanical response of the cornea subjected to a non-contact air-jet tonometry diagnostic test represents an interplay between its geometry, the corneal material behavior and the loading. The objective is to study this interplay to better understand and interpret the results obtained with a non-contact tonometry test. A patient-specific finite element model of a healthy eye, accounting for the load free configuration, was used. The corneal tissue was modeled as an anisotropic hyperelastic material with two preferential directions. Three different sets of parameters within the human experimental range obtained from inflation tests were considered. The influence of the IOP was studied by considering four pressure levels (10–28 mmHg) whereas the influence of corneal thickness was studied by inducing a uniform variation (300–600 microns). A Computer Fluid Dynamics (CFD) air-jet simulation determined pressure loading exerted on the anterior corneal surface. The maximum apex displacement showed a linear variation with IOP for all materials examined. On the contrary, the maximum apex displacement followed a cubic relation with corneal thickness. In addition, a significant sensitivity of the apical displacement to the corneal stiffness was also obtained. Explanation to this behavior was found in the fact that the cornea experiences bending when subjected to an air-puff loading, causing the anterior surface to work in compression whereas the posterior surface works in tension. Hence, collagen fibers located at the anterior surface do not contribute to load bearing. Non-contact tonometry devices give useful information that could be misleading since the corneal deformation is the result of the interaction between the mechanical properties, IOP, and geometry. Therefore, a non-contact tonometry test is not sufficient to evaluate their individual contribution and a complete *in-vivo* characterization would require more than one test to independently determine the membrane and bending corneal behavior.

## Introduction

The shape of the cornea is the consequence of the equilibrium between its mechanical structure and the forces acting upon it. The mechanical behavior of the cornea depends on its geometry (thickness, curvature and topography) and material properties, which in-turn relies on the microstructure of the stroma. The combination of high-speed photography (Scheimpflug imaging) of corneal images and dynamic bidirectional applanation technologies has been recently proposed as a new potentially useful method for evaluating the mechanical behavior of the cornea [1,2]. These devices, known as non-contact tonometers, record the corneal motion while an air pulse forces the deformation, and report the deformation amplitude of the cornea, the applanation length and the corneal velocity over time [1,3]. Likewise, intraocular pressure (IOP) and corneal apical pachymetry data, e.g. corneal central thickness (CCT), are also provided [3].

To date, a limited number of studies evaluating the clinical application of this device have been performed [1,3–8]. Huseynova et al [4] found a low but moderate correlation of IOP with deformation amplitude of the cornea (Pearson’s correlation coefficient *ρ* = -0.360, *p*<0.0001), applanation time (*ρ* = -0.540, *p*<0.0001) and applanation velocity (*ρ* = -0.118, *p*<0.0001). Unfortunately, they did not report any correlation of corneal central thickness (CCT) with the deformation amplitude of the cornea. In a recent review, Roberts [9] states that IOP is the strongest predictor of corneal deformation amplitude, followed by corneal stiffness, and CCT being the less influential along with the curvature. Valbon et al [8] reported a weak correlation between the highest concavity-time (the time at which the highest concavity of the cornea is reached) and age for healthy eyes. In addition to the work performed on healthy eyes, Faria-Correia and co-authors [7] have found that ocular hypertension in pressure-induced stromal keratopathy is associated with lower deformation response. However, there is no scientific evidence showing the relationship between the analysis of the response to the air-puff and the parameters characterizing the mechanical properties of corneal tissue.

Patient specific geometrical models are useful for performing a diagnosis test. This gives doctors the opportunity of improving their diagnosis by relying on real patient data, rather than in a generalized statistical atlas. Late eyeball models already use patient-specific models based in Zernike interpolation and point cloud data reconstruction obtained from a topographer in order to represent a specific cornea for each patient [10,11,12]. In this work, an automatic methodology that generates a patient-specific corneal model is used [13].

Besides, the *in-vivo* human cornea is a porous tissue with high water content (approximately 80% of the corneal weight is due to water). Among the five layers that constitute the cornea, the stroma forms about 90% of the thickness and is composed of long collagen fibers embedded in a ground substance mainly formed of proteoglycans and water. Collagen fibers lie parallel to the corneal surface and are orthogonally disposed along the superior-inferior and nasal-temporal directions whereas they are predominantly circumferential near the limbus. This microstructure and the different distributions of collagen fibers give the corneal tissue an anisotropic mechanical behavior. Therefore, the constitutive material behavior of the cornea was considered as anisotropic hyperelastic accounting for the two families of collagen fibers present in the eye [14,15,16].

A finite element (FE) analysis of a non-contact tonometer is performed. This simulation was not intended to reflect any commercial device, but only to replicate a typical evaluation test. In this regard, the characteristic of the test, i.e., peak pressure of the air-puff, and the location and duration of the air pulse, were set in order to emulate a general non-contact tonometer, since the aim of the study is to better understand the relation that the corneal material behavior, IOP, and pachymetry have with the deformation that the cornea experiences when subjected to this type of diagnosis test [9,18].

In order to achieve this objective, different sets of experiments were designed. The influence of the IOP has been studied by considering four pressure levels (10 mmHg, 12 mmHg, 19 mmHg and 28 mmHg) and three different levels of corneal stiffness (low (material A), intermediate (material B) and large (material C) stiffness) taking into account the material ranges reported in the literature. In addition, the relation between the CCT and the maximum apical displacement during an air puff diagnostic test was studied by varying the CCT from 300 microns to 600 microns along with the corneal tissue stiffness variation (material A, material B and material C) and three levels of IOP (10 mmHg, 19 mmHg, 28 mmHg).

As final goal, this study seeks to gain a better understanding of the coupling existing between the aforementioned parameters in order to better interpret the results obtained with a non-contact tonometry test.

## Material and Methods

### Corneal Geometry and Patient-Specific Corneal Finite Element Model

The right healthy cornea of a 25-year man was considered in the study (data shown in Table 1). The individual in this manuscript has given written informed consent (as outlined in PLOS consent form) to publish these case details. All procedures were carried out under project license of POPCORN project approved (31^st^ October, 2013) by the Ethics Committee of the Research of the University of Alicante (Comité de Ética de la Investigación de la Universidad de Alicante, CEUA). The corneal topographic map reconstructed using a Pentacam system (Oculus Optikgeräte GmbH, Germany) showed the conventional bow-tie pattern, without significant asymmetry. The main parameters were: a central corneal thickness (CCT) of 585 microns and a Goldmann IOP of 12 mmHg.

**Table 1:** Right healthy eye data provided by the Pentacam.

**IOP**	12 mmHg	
**Apex Pachymetry**	585 microns	
**Min Pachymetry**	583 microns	
**Corneal Volume**	63.5 mm^3^	
	**Anterior Surface**	**Posterior Surface**
**Corneal Astigmatism**	0.4D	0.3D
**Corneal Asphericity**	-0.22	-0.12
**Average Radius**	8.08 mm	6.64 mm

**Fig 1:**
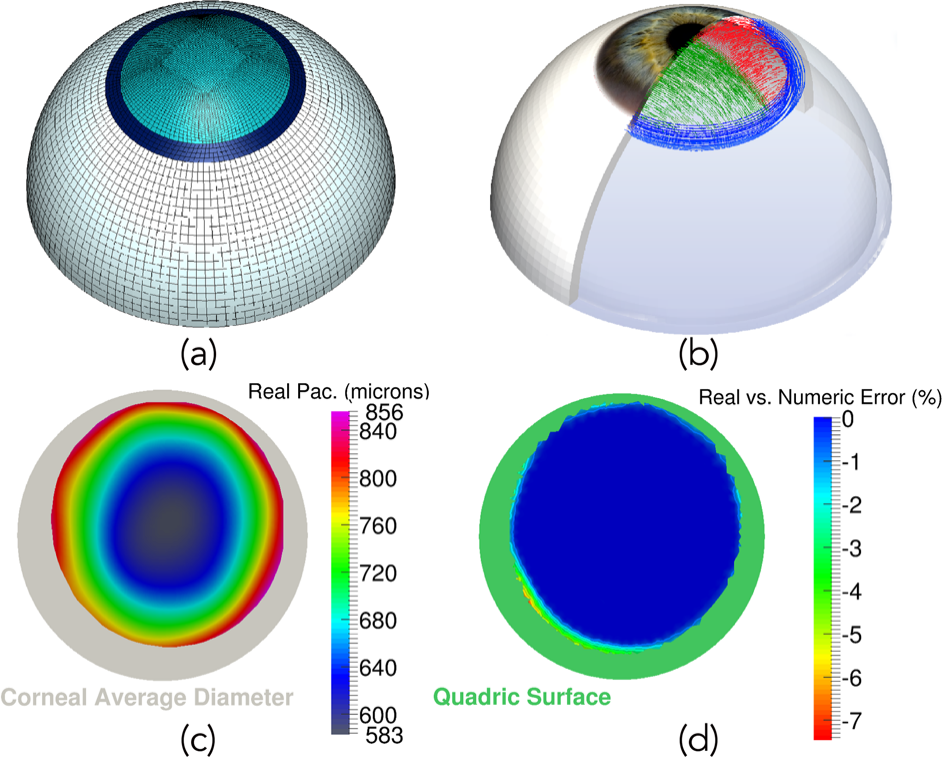
Three-dimensional eyeball model, collagen fiber distribution, pachymetry and patient-specific validation. a) Finite element model of the eye. Light blue mesh corresponds to the patient-specific cornea obtained by means of the Pentacam system; the limbus is shown as dark blue area, whereas sclera is white; b) Corneal Collagen Fiber Distribution (nasal-temporal fibers in green and superior-inferior fibers in red) and Limbo Fiber orientation (blue fibers distributed circumferentially); c) Actual patient’s pachymetry given by the Pentacam topographer (grey area shows the average corneal size considered for the 3D FE model); d) Error difference (%) between the actual patient’s pachymetry and the pachymetry of the numerical model. Blue values represent a truly patient-specific corneal thickness (green area belongs to the quadric surface necessary to extend corneal data to the average desired diameter).

A three-dimensional finite element model of the anterior half ocular globe geometry, which accounts for three different parts: the cornea, the limbus and the sclera, was considered (Fig. 1a). A methodology for constructing patient specific corneal models has been used [13] which allows building the patient specific model of the cornea by using the topography of the anterior surface of the cornea and the pachymetry data [10,11,12]. Real data was kept at those points where topographical data was available (see the pachymetry map in Fig. 1c), whereas a quadric surface was used to complete the cornea up to a mean diameter of 12 mm [13,14] (see the grey area in Fig. 1c). In order to further demonstrate the patient-specific characteristics of the model, the difference between the patient’s and numerical pachymetry used in the FE model is shown in Fig. 1d. As it can be observed, the patient-specific is fully achieved in those points where data were known (see blue area belonging to 0% error difference), obtaining only a maximum error difference of a 7.5% at the joint between the quadric surface (see green area in Fig. 1d) and the real surface. This error is related to the smoothing algorithm used to joint both surfaces in order to avoid numerical problems due to surface discontinuity [13]. In addition, a 25 mm, in average, diameter sphere was considered for the sclera, whereas the limbus is a ring linking both, sclera and cornea. Axial displacements and rotations were restrained at the bottom surface of the sclera [19,20].

A mesh composed of 13,425 quadratic full integration mixed formulation solid elements and 62,276 nodes was used to perform the simulations. To test the quality of the mesh used for the calculations, a sensitivity analysis was performed. Table 2 shows the change in the apical displacement (Δ*U*) and maximum principal stress (Δ*PS*) in the cornea for different mesh densities. The results show that for a mesh size above approximately 60000 nodes, the changes in the apical displacement are less than a 0.05%, whereas for the maximum principal stress is less than 2.0%, demonstrating the adequacy of the used mesh.

**Table 2:** Mesh sensitivity analysis.

Number of Nodes	Relative Change in apical displacement (%)	Relative Change in maximum principal stress (%)
**27800**	—	—
**50304**	0.4	100
**62276**	0.05	20
**139920**	0.05	1.5

### Constitutive model for eyeball’s tissue

The cornea was considered as an anisotropic [1,21] hyperelastic material with two preferred material directions [15,22] (see in Fig. 1b) modeled using a Gasser-Holzapfel-Ogden’s (G-H-O) constitutive equation (1) [16].

$$\begin{array}{l} U=C_{10}({\bar{I}}_{1}-3)+\frac{1}{D}\left(\frac{{\left(J^{el}\right)}^{2}-1)}{2}-ln(J^{el})\right)+\frac{k_{1}}{2k_{2}}\sum_{\alpha =1}^{N}\left(exp\left[k_{2}{\langle E_{\alpha }\rangle }^{2}\right]-1\right)\\ \langle E_{\alpha }\rangle \overset{def}{=}\kappa ({\bar{I}}_{1}-3)+(1-3\kappa )({\bar{I}}_{4(\alpha \alpha )}-1) \end{array}$$ (1)

where ${\bar{I}}_{1}$ is the first modified strain invariants of the symmetric modified right Cauchy-Green tensor, *J* ^*el*^ is the elastic volume ratio, and the pseudo-invariant ${\bar{I}}_{4(aa)}$ is the square of the stretch along the fiber directions, with N the number of families of collagen fibers. The parameter *k* ∊[0,1/3] describes the level of dispersion along fiber directions. A value of k = 0 implies that fibers are perfectly aligned (no dispersion), whereas k = 1/3 indicates that fibers are randomly distributed and the material behaves isotropically. Equation (1) assumes that all families of fibers have the same mechanical properties as well as the same dispersion. Another basic assumption of the model is that collagen fibers can only support tension, since they would buckle under compressive loading. Thus, the anisotropic contribution to the strain energy function appears only when the strain,*E*_*α*_, is positive, i.e.,*E*_*α*_>0.

**Fig 2:**
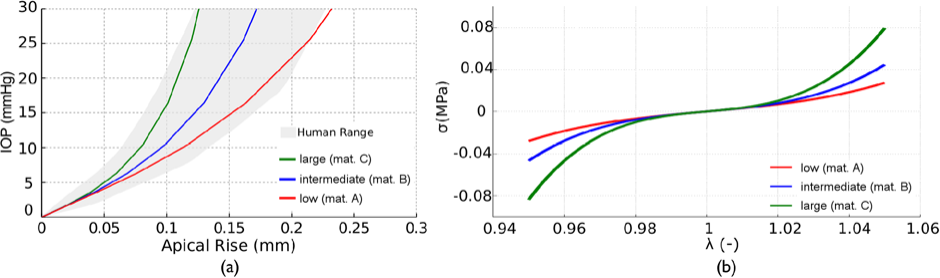
Human corneal response of the constitutive model. a) IOP (mmHg) vs. Apical Rise (mm). Human range (grey shadow) obtained from inflation test in human corneas [23,24]. Colored lines correspond to inflation response for the three material selected for the numerical simulation: low(material A)—red, intermediate(material B)—blue, large(material C)—green); b) Uniaxial stress-stretch behavior for the three studied materials.

Three sets of material parameters associated with: low (material A), intermediate (material B) and large (material C) stiffness, were considered in our simulations (Table 3). These sets of parameters span the experimental IOP (mmHg)—Apical Rise (mm) curves response obtained from inflation tests on human corneas [23, 24,25] (see in Fig. 2).

**Table 3:** Material parameters for cornea, limbus, and sclera.

Cornea and Limbus						
Material	Type	C10 (MPa)	D(MPa^-1^)	k1 (MPa)	k2 (-)	*k* (-)
**A**	H-G-O	0.05	0.0	25.0	2490	0.33329
**B**	H-G-O	0.05	0.0	60	2490	0.33329
**C**	H-G-O	0.05	0.0	130.9	2490	0.33329

Sclera				
Type	D	C10 (MPa)	C20 (MPa)	C30 (MPa)
Yeoh	0.0	0.81	56.05	2332.26

The viscoelastic behavior of the tissue was neglected since a very fast applied load, as the case of the air-puff, will result in an almost pure elastic response during the loading [26], and corneal hysteresis is observed only during the unloading phase of the air-puff load only affecting the recovery response of the cornea (which is not the objective of this study). Limbus’ material parameters have been assumed identical as for the cornea.

The sclera was considered as an isotropic hyperelastic material (Table 3) and was modeled using the Yeoh’s constitutive model (Equation 2) [27, 28].

$$U=\sum_{i=1}^{3}C_{i0}{\left({\bar{I}}_{1}-3\right)}^{i}+\sum_{i=1}^{3}\frac{1}{D_{i}}({\left(J^{el}-1\right)}^{2i})$$ (2)

### Non-contact tonometer simulation

**Fig 3:**
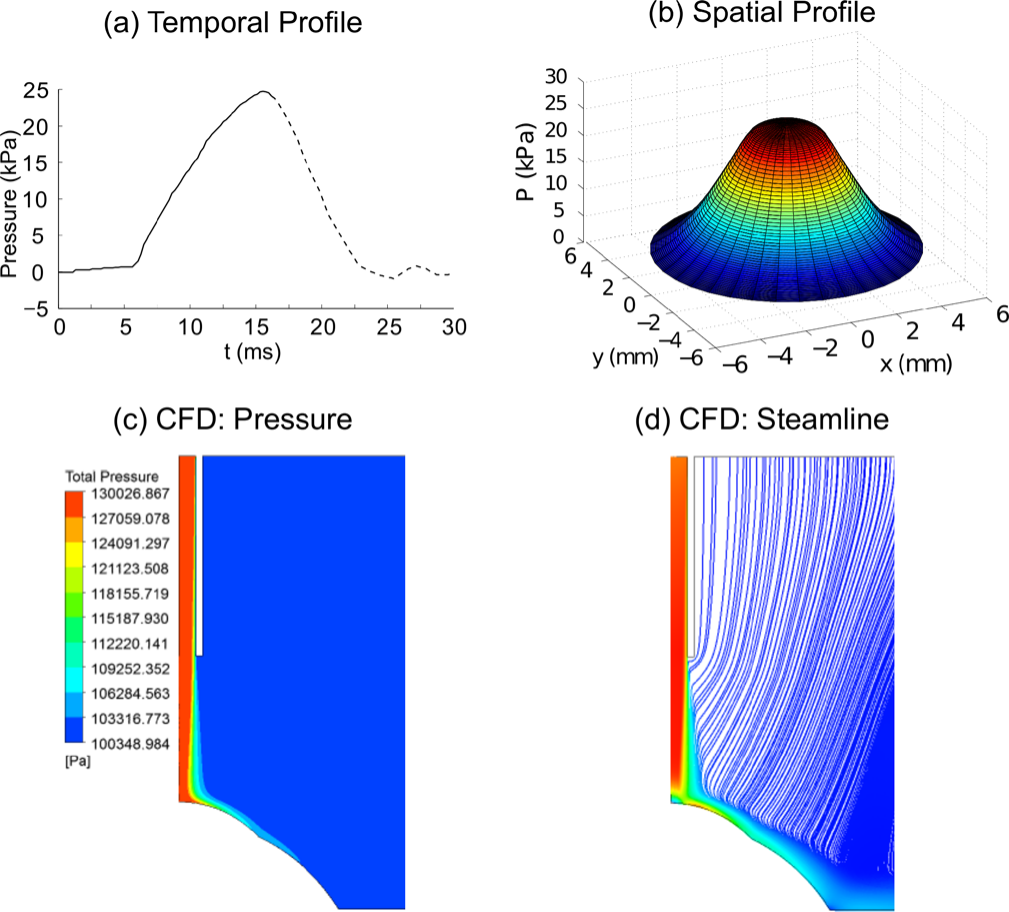
Non-contact tonometer air-puff loading and CFD results. a) Temporal pressure profile applied on the center of the cornea (corneal apex region). Solid black line represents the temporal profile used in the simulations. Dashed black line was no considered since only the maximum displacement of the corneal apex was studied; b) Spatial profile of pressure applied on cornea obtained with the CFD simulation shown in c) and d); c) Symmetrical pressure profile obtained from the CFD simulation; d) Symmetrical velocity streamline plot result from the CFD simulation.

Since the patient’s eye is subjected to the IOP when the topographical data is acquired, the prior step to simulating the non-contact tonometry test is the identification of the initial stress-free configuration of the eye. An iterative zero pressure algorithm was applied in order to obtain the initial free-stress configuration of the eye [13,17,29] in each simulation. The algorithm applies an IOP to an initial free-stress geometry ($X_{init}^{k}$) for obtaining the first deformed configuration ($x_{def}^{k}$). Once the pressurization ends, the difference error between the deformed configuration and the topographer’s geometry ($X_{ref}^{0}$) is computed ($E^{k}=x_{def}^{k}-X_{ref}^{0}$). If the infinite norm of the error ($e^{k}=|E^{k}{|}_{\infty }=\max(E_{i}^{k})$) is higher than a given tolerance (*ε*), a new initial configuration is computed ($X_{init}^{k+1}=X_{init}^{k}-E^{k}$). Otherwise, the initial configuration obtained is such that when it is pressurized to IOP, it achieves the measured configuration. For the simulation of the non-contact tonometry test, the air-puff was assumed as a metered collimated air pulse with a peak pressure of 25 kPa (~180 mmHg) and 30 ms duration, with a profile given in Fig. 3a (personal communication with Oculus). The air-puff spatial pressure (see Fig. 3b) was obtained from a CFD simulation performed with the commercial software ANSYS (see Fig. 3c-d) in order to load the corneal as close to reality as possible. As shown in Fig. 3c-d, the desired peak pressure applied to the cornea lies on an approximated circular area of 3 mm in diameter.

A total of 41 numerical experiments were performed to study the influence of: i) IOP, ii) corneal thickness, and iii) material behavior (stiffness), on the maximum corneal displacement. Numerical simulations have been performed on the finite element software ABAQUS (Dassault Systemes), using a conventional personal computer (8-cores i7–4770 3.4 GHz, 8 GB RAM) requiring a computation time of approximately 45 minutes to perform a full simulation. Visualizations of the results carried out with the software ParaView (Kitware Inc. and Los Alamos National Laboratory) [30].

## Results

**Fig 4:**
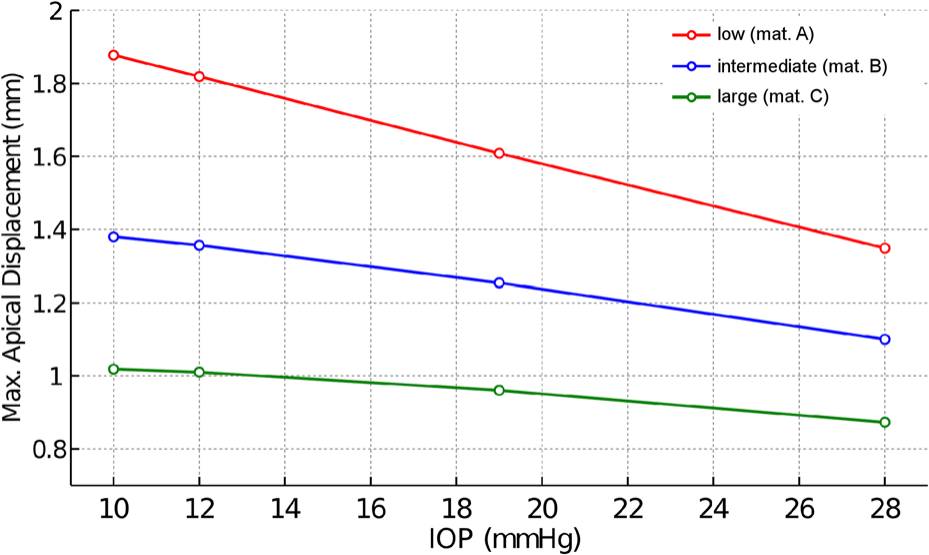
Displacement—Pressure response of the corneal apex. Vertical displacement of the corneal apex (mm) as a function of IOP (10, 12, 19 and 28 mmHg) for the three material models: low (material A), intermediate (material B), large (material C) stiffness.

Fig. 4 shows the deformation amplitude of the corneal apex for different IOP and different corneal material response. The corneal deformation following the air pulse varies linearly with IOP, with larger displacements corresponding to lower IOP.

**Fig 5:**
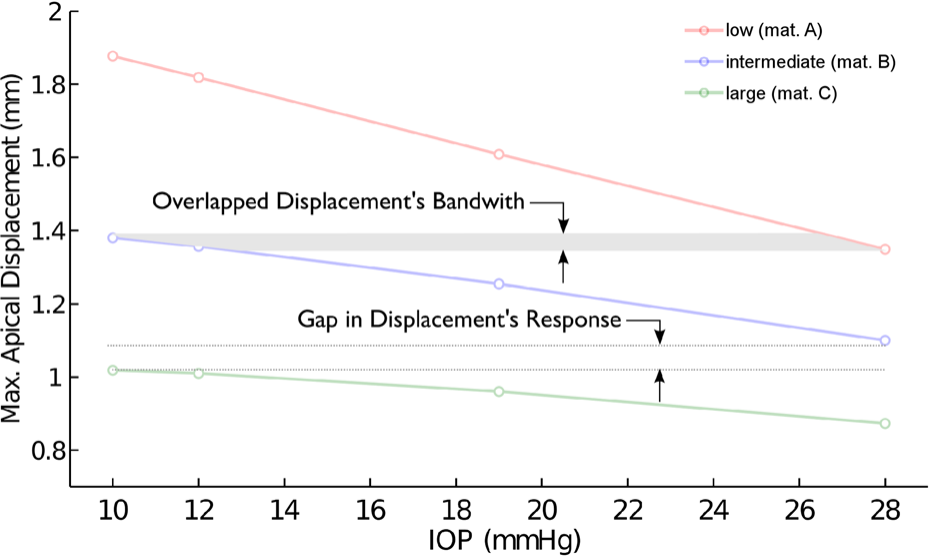
Displacement—Pressure: response overlapping. Overlapping zone in the corneal response (grey zone) where different combinations of IOP and material lead to the same displacement.

Fig. 5 shows that different combinations of material parameters (within the reported human range [23,24]) and IOP could produce the same apical displacement (see the overlapping area in Fig. 5).

**Fig 6:**
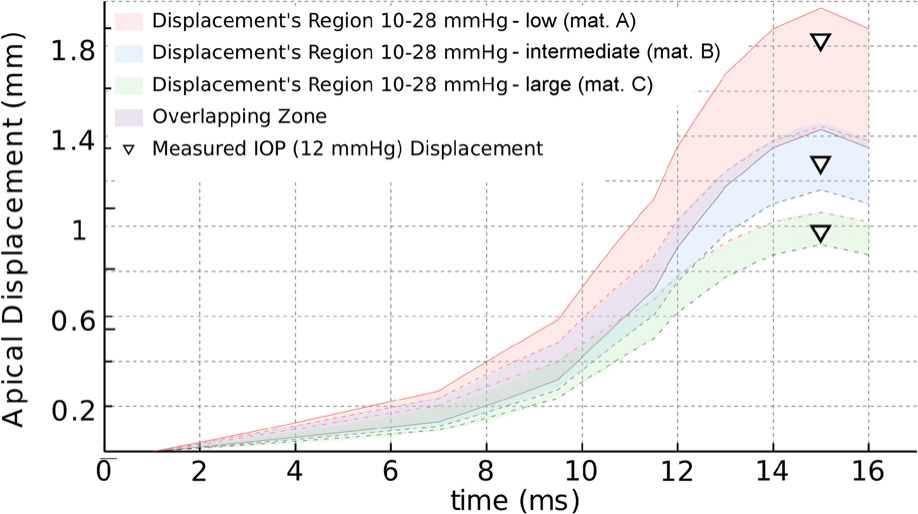
Displacement—Time response of the corneal apex. Time course of the apex displacement for the conducted simulations. Displacement’s region 10–28 mmHg (mat. A) (red colored area) are the results for low stiffness material (A) for all three different IOP (10, 19 and 28 mmHg); Displacement’s region 10–28 mmHg (mat. B) (blue colored area) are the results for intermediate stiffness material (B) for all three different IOP (10, 19 and 28 mmHg); Displacement’s region 10–28 mmHg (mat. C) (green colored area) are the results for large stiffness material (C) for all three different IOP (10, 19 and 28 mmHg). Different overlapping zones, at different loading time, can be observed in figure. Inverted triangles correspond to simulations performed with the real IOP (12 mmHg) and the three different corneal material models.

This fact is better demonstrated in Fig. 6 showing the temporal evolution of the displacement of the apex for three values of IOP (10 mmHg, 19 mmHg and 28 mmHg) and the three analyzed corneal mechanical properties (only half of the time history is shown). This figure also shows the maximum apical corneal displacement corresponding to the patient’s IOP = 12 mmHg, depicted as inverted triangles, for all three materials. An overlapping zone can be observed for two of the chosen materials: the softest material coupled with the highest IOP, and the intermediate stiffness material with the lowest IOP (see overlapping zone in Figs. 5 and 6). On the contrary, there is a gap zone between the most rigid material and the intermediate stiffness material (see Figs. 5 and 6). Hence, different combinations of IOP and material stiffness may lead to the same maximum displacement of the corneal apex indicating the existence of a coupling between the effect of corneal material behavior and the IOP.

**Fig 7:**
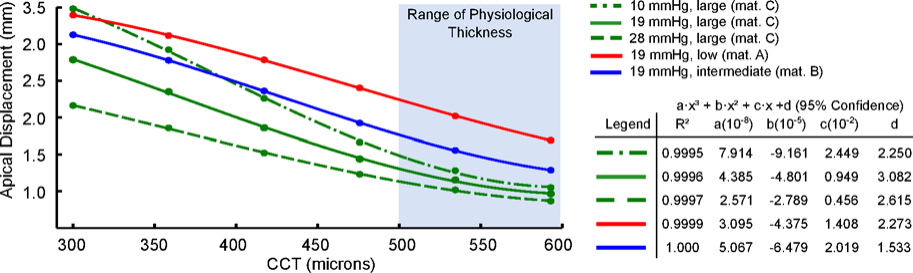
Displacement of the corneal apex (mm) as a function of the corneal thickness (CCT). Patient’s pachymetry was constantly decreased for the simulations. Results show a cubic relation between displacement and pachymetry (CCT) when the material was fixed (large stifnees material—C) and three levels of IOP were considered: 10 mmHg (dotted-dashed green line), 19 mHg (solid green line), and 28 mmHg (doted green line). Results also show a cubic relation between displacement and pachymetry (CCT) when the IOP was kept at 19 mmHg and the three corneal stiffnesses were considered: low (material A) solid red line, intermediate (material B) solid blue line, and large (material C) solid green line. The right panel shows the accuracy of the fit (minimum mean squares) and the constants of the cubic polynomial.

Fig. 7 shows the effect of corneal thickness on the maximum apical displacement. In general, as the thickness decreases below 500 microns, the maximal corneal displacement increases rapidly, reaching values up to three times larger for corneal thickness below 400 microns. A closer analysis of the results show a cubic relationship (right panel on Fig. 7) between the maximum apex displacement and the corneal. This cubic relationship was found independently of either the material stiffness or the IOP. For a given material stiffness, the influence of corneal thinning is more prominent as the IOP decreases (green lines in Fig. 7). On the contrary, this effect seems to be less acute when the corneal stiffness decreases while IOP remains constant (solid lines in Fig. 7). This figure also shows the interplay between the corneal geometry, the corneal mechanical properties, and the IOP by which different combinations of these variables could lead to the same maximum apical displacement.

**Fig 8:**
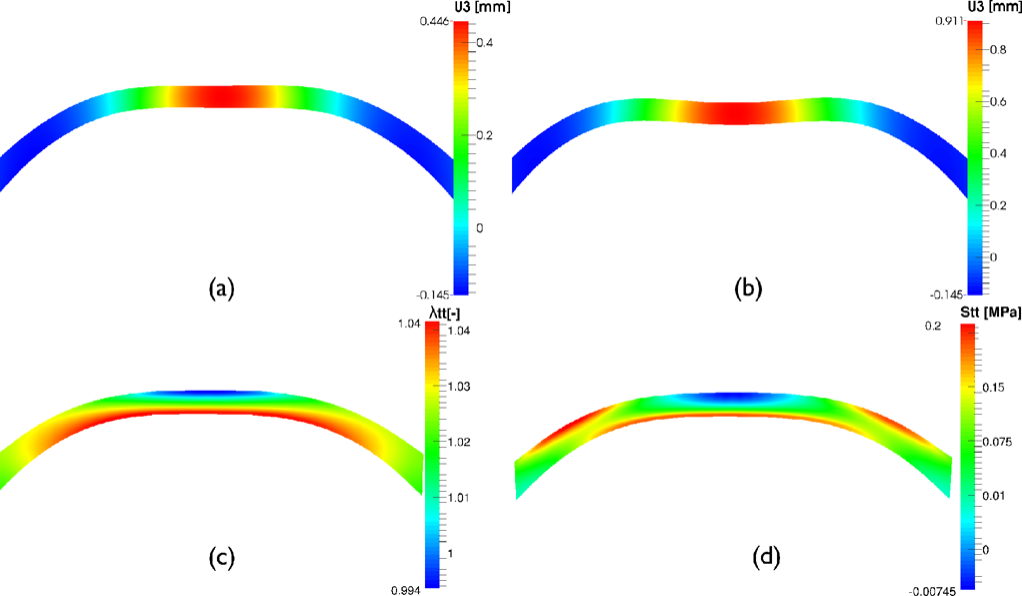
Corneal response in the meridional plane (FE results). Meridional cutting plane of the cornea. a) Displacement field along the optical axis at first applanation time (mm), b) Vertical displacement field at high concavity time (mm), c) Circumferential logarithmic strain field (-) at first applanation time, d) Hoop stress field (MPa) at first applanation time (spherical coordinate system). c) and d) show the bending mode of deformation at which the cornea is subjected during a non-contact tonometry test. Results correspond to IOP = 19 mmHg and material C.

Fig. 8 shows the deformed shape of the cornea’s central section at the instant of first applanation (Fig. 8a) and of highest concavity (Fig. 8b). In addition, the logarithmic hoop strain and the hoop Cauchy stress at highest concavity time are depicted in Fig. 8 c-d (plotted on the non deformed configuration for a more clear representation). The figure shows that during the air-puff the cornea experiences bending and, therefore, the anterior surface works in compression (see blue zone in Fig. 8 c-d) whereas the posterior surface works under tension (see red zone in Fig. 8 c-d). This means that the collagen fibers in the anterior surface do not contribute to load bearing after the first applanation, since they only work under traction (as in the case of a rope). Hence, at those points the material response depends only on the compressive behavior of the stroma.

**Fig 9:**
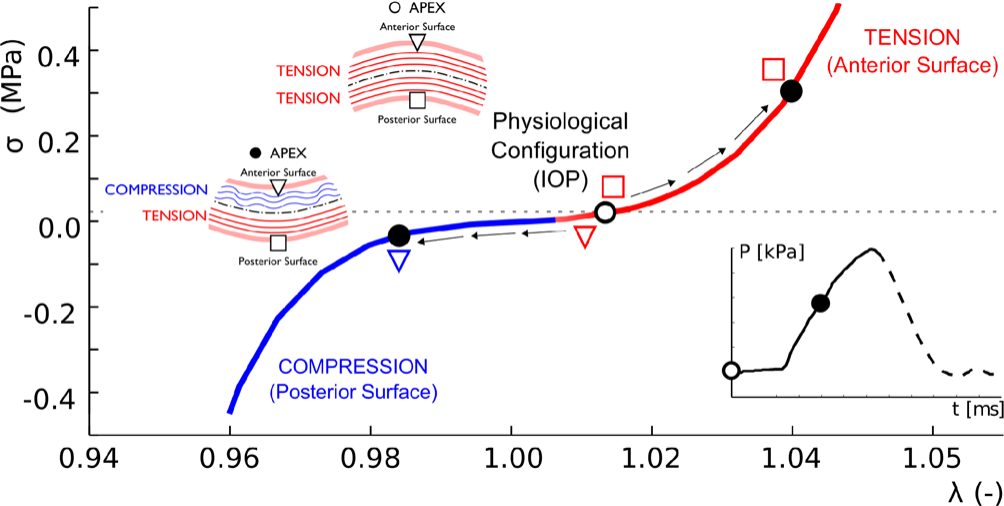
Stress strain response of anterior and posterior apical points during non-contact tonometry. Normal Cauchy Stress vs. stretch path along the meridional direction followed by two points on the anterior and posterior surface of the cornea during air-puff for an IOP = 19 mmHg and the stiffest material (C). Blue color is associated with compression whereas red color is associated with tension. At the physiological configuration when the eye is subjected to IOP (open circle at the beginning of the air jet profile, shown in the inset) the cornea only experiences traction (membrane tensional state). As the air-pulse progresses (black filled circle in the pressure profile inset), the anterior corneal surface (inverted open triangle) experiences compression (*λ*<1) whereas, the posterior corneal surface (open square) experiences a larger tensional stress (*λ*>1).

Results from Fig. 8 are demonstrated further in Fig. 9, where the stress-stretch path followed by a point located at the apex (inverted triangle) and its mirror image on the posterior surface (square) during air-puff are depicted. At the beginning of the test (empty circle on the stress-stretch curve) both points are subjected to traction (*σ>0*, *λ*>1) due to the effect of the IOP. However, during the air-pulse, the state of stress in the anterior surface changes from a traction state to a compression state (see the trajectory of the inverted triangle in Fig. 9), whereas the posterior surface remains in traction (open red square).

## Discussion

Modern clinical methods for evaluating the biomechanics of the cornea are based on studying the deformation of the cornea when subjected to the action of a metered collimated air pulse with symmetrical configuration applied at the apex of the cornea [1,2]. This *in-silico* study conducted on a patient-specific patient eye geometry seeks to gain a better understanding of the interplay between the structural characteristics of the cornea, its material behavior, and loading on the mechanical response of the cornea when subjected to an air-puff.

For corneal stiffness within the observed physiological range, the maximal corneal displacement was found to follow a linear relation with IOP. In addition, the range of the maximum apical displacement from the numerical simulation was in good agreement with the maximum apical displacement reported in a study with 89 healthy eyes, i.e., 0.78–1.26 mm, using the CorVis ST system. In the study by Valbon et al [8] the IOP and CCT ranged between 7 and 32 mmHg and between 463 and 605 microns respectively. Huseynova et al [4] studied the influence of IOP and CCT on the different markers provided by the CorVis ST system. These authors found that the deformation amplitude of the cornea varied between 0.9 and 1.3 mm in the subgroup of the analyzed sample (III group) having a corneal-compensated intraocular pressure (IOPcc) between 18 and 21 mmHg and a CCT between 555 and 600 microns. These experimental results on healthy eyes are also within the results reported in our study, confirming the soundness of our model for simulating the corneal response under the action of an air-puff.

According to our simulations, the maximum displacement of the apex due to the air pulse varies linearly with IOP for all corneal stiffness considered, with the largest displacement corresponding to the lowest IOP value. This result was consistent with previous results [4], where a moderate negative correlation between IOPcc and maximum corneal deformation was also found (*r* = -0.362, *p*<0.0001). A similar conclusion was stated in the experimental study conducted by Kling and Marcos [31]. Hence, the results of the simulations suggest that the mechanical corneal response to an air pulse pressure varies linearly with IOP.

Figs. 5 and 6 illustrates that the right combination of corneal stiffness and IOP may result in the same maximal corneal deformation. Depending on the stiffness of the corneal tissue (characterized by a different set of material model parameters) and the IOP of the examined eye, an overlapping zone could exist. Therefore, it is not possible to distinguish between individual effects (IOP and material stiffness) without knowing the characteristic of one of them *a-priori*, e.g. Cornea’s stiffness.

As shown in Fig. 7, the apex displacement during a non-contact tonometry showed a cubic relationship with corneal thickness. For a corneal thickness within the physiological range (500–600 microns), the maximal corneal displacement ranged withing the reported clinical range (0.7–1.3 mm). However, as the thickness decreases below 500 microns, the maximal corneal displacement increases rapidly. These corneal response at low CCT values could correspond to an extreme LASIK intervention or an advanced ectasia (e.g. Keratoconus disease) in which the local corneal thinning could lead to a larger apical displacement as compared to healthy patients presenting a regular and smooth pachymetry.

It should also be pointed out that, when the cornea is under the action of the IOP, the state of stress of the cornea corresponds to a pure traction membrane state, which means that the full cornea works in tension when it is subjected to its physiological IOP (i.e. no bending effects exists and it behaves as expected according to the shells and laminates theory), as shown in Fig. 9. However, during air puff, the cornea experiences bending and, therefore, the anterior surface goes from a traction state of stress to a compression state of stress whereas the posterior surface works in tension (see Fig. 9). This implies that collagen fibers in the anterior surface do not contribute to load bearing during the total duration of the air-puff, relying in this cases on the mechanical properties of the matrix. This non-physiological situation implies that the biomechanical characterization using an air pulse pressure loading accounts for the contribution of the collagen fibers only partially (only the posterior part of the cornea), contrary to the case of an inflation test where the cornea works under tension all the time. Therefore, the mechanical response characterized by non-contact tonometry represents a combination of the mechanical behavior of the cornea under traction (associate with the collagen fiber network) and, but not less important, the mechanical behavior under compression of the stroma. This is important since the state of the art mechanical testing of the cornea accounts for the mechanical response under tension only [23,24,27,32].

In addition to the corneal stiffness and IOP, the clinical study by Huseynova et al [4] reported the CCT as another parameter with significant influence on the corneal response analyzed by non-contact tonometry using the CorVis ST system. In particular, these authors found significant differences in the first applanation time and radius of curvature at highest concavity between central corneal thickness subgroups for each IOPcc group that was studied (*p*<0.0001). Numerical results from our study also suggest this. Our results show a cubic relationship between the maximum apical displacement and the corneal thickness. This cubic dependence obeys to the bending deformation induced in the cornea during the action of the air-pulse, as expected in a thin shell subjected to bending. This cubic dependency helps to explain the increment in corneal displacement on patients that have undergone LASIK surgery and whose cornea has suffered a significant reduction of thickness and curvature. A similar situation is found in patients with narrow diseased corneas affected by ectasia.

Hence, the corneal response to an air puff is influenced by the mechanical properties of the cornea, the IOP, and topology (i.e., CCT and curvature). Therefore, analysis based on the evaluation of corneal response to an air pulse pressure, provides a response of the combined contribution of these effects, without being able to uncouple the precise contribution of each factor. By this, the corneal mechanical properties cannot be assumed to be directly related to the parameters defining the corneal response to the air pulse. This fact has been also demonstrated by Glass et al [33] who have developed and validated a viscoelastic model to illustrate how changing viscosity and elasticity may affect corneal hysteresis (CH), concluding that low CH could be associated with either high elasticity or low elasticity, depending on the viscosity.

A final comment is devoted to the limitation of this study. Corneal material has been assumed as hyperelastic, neglecting the intrinsic viscoelastic behavior of the tissue. However, since we were only interested in the maximum apex displacement, attained during the pressure rising phase of the air puff, and taking into account that a very fast load will result on an almost pure elastic response [26], it can be assumed that neglecting a corneal viscoelastic behavior would not significantly affect the results of study. Viscoelasticity may, however, have significant importance on the relaxation phase of the cornea after the air-puff stops. In addition, the study shows only results on one patient, and therefore, statistics regarding the influence of the patient specific eye geometry and IOP has not been computed yet. We are currently applying the presented *in-silico* methodology to a larger set of healthy and pathological patients in order to determine the impact of patient-to-patient geometric variability and IOP on maximum corneal displacements and other proposed mechanical biomarkers.

In conclusion, the proposed *in-silico* methodology allows computing a sensitivity analysis of the mechanical properties of the corneal tissue, the IOP and the geometry of the cornea on the corneal deformation of patient specific geometric eye models. This type of analysis is not possible with standard non-contact tonometry devices since it has been demonstrated that they measure the combined contribution of all these factors on the corneal response. Thus, a cornea with high stiffness and low IOP may show the same deformation response as a cornea with low stiffness and high IOP. In addition, systems based on non-contact tonometry for characterizing the corneal biomechanics evaluates the mechanical response of the cornea under bending, whereas the corneal response to variations of the IOP depends on a pure membrane behavior of the cornea, a condition that is only achieved in biaxial or inflation loading. These results indicate that a complete *in-vivo* corneal mechanical characterization would require more than one test in order to determine the membrane and bending behavior of the cornea independently.
